# Acute Ovarian Insufficiency and Uterine Infarction Following Uterine Artery Embolization for Postpartum Hemorrhage

**DOI:** 10.23937/2378-3656/1410040

**Published:** 2015-06

**Authors:** Sarah Z. Elsarrag, Abigail R. Forss, Susan Richman, Sana M. Salih

**Affiliations:** 1 Department of Obstetrics and Gynecology, Divisions of Reproductive Endocrinology and Infertility, University of Wisconsin, USA; 2 Department of Obstetrics and Gynecology, Yale University School of Medicine, USA; 3 County Obstetrics & Gynecology Group, New Haven, USA

**Keywords:** ovarian insufficiency, infertility, fertility preservation, menopause, postpartum hemorrhage, pelvic artery embolization, uterine infarction

## Abstract

Uterine artery embolization for intractable postpartum hemorrhage saves lives while preserving fertility. The procedure-related risks of uterine infarction and ovarian insufficiency are rare. A primparous patient underwent bilateral internal hypogastric artery embolization to control severe postpartum hemorrhage following primary cesarean section. The bleeding continued, and a repeat aortogram demonstrated significant filling of the uterus from an anomalous proximal take off of the right uterine artery and from the left ovarian artery. Further embolization was required to control the bleeding. The patient developed acute primary ovarian insufficiency within two weeks of the procedure and subsequently presented with uterine infarction necessitating hysterectomy. This case demonstrates the increased risk of acute ovarian insufficiency and uterine infarction following uterine artery embolization for postpartum hemorrhage in the settings of aberrant pelvic vasculature.

## Introduction

Uterine artery embolization (UAE) for intractable postpartum hemorrhage (PPH) saves lives while preserving fertility [1,2]. The procedure-related risks of uterine infarction and ovarian insufficiency are rare. To date, two cases of fatal sepsis following UAE for uterine fibroids have been reported [3,4]. A total of seven cases of uterine infarction necessitating hysterectomy have also been described; two of these cases occurred following UAE for PPH, while five cases occurred following UAE for uterine fibroids [5-15]. The effect of UAE on ovarian reserve remains controversial. Attempts to induce ovarian ablation via selective ovarian artery embolization in patients with severe endometriosis that fail medical treatment have not been successful in our institution. Inadvertent ovarian artery embolization leading to ovarian failure following UAE for PPH has, however, been described [16]. A randomized comparison measuring AMH levels following UAE for fibroids indicated that AMH level, which is a measure of ovarian reserve, is decreased when compared to surgical hysterectomy [16,17]. Others have reported that UAE does not appear to impair fertility when treating fibroids for women of childbearing age, but women older than 45 years of age are at increased risk for permanent ovarian failure due to their already decreased ovarian reserve [18,19]. Here, we report a case of acute ovarian insufficiency occurring within two weeks of UAE for PPH, most likely due to anomalous pelvic vasculature with large uterineovarian arteries anastomosis. While variations in pelvic vasculature have been described [20], they have not been reported in association with acute ovarian insufficiency.

## Materials and Methods

An unremarkable primary elective cesarean section with the delivery of healthy twin male infants was performed in a 29-year-old nulliparous female at term. Delivery was complicated by severe PPH and coagulopathy due to uterine atony. Management of the PPH bleeding was not effective, despite uterine compression, massage, and treatments with oxytocin, methergine, carboprost, misoprostol, erythrobiotin, DDAVP, Aminocaproic acid, and vitamin K. Being a Jehovah’s Witness, the patient refused blood transfusion but agreed to receive cryoprecipitate. She was given crystalloids, Hetastarch, albumin, and cryoprecipitate infusions. The patient was counseled for UAE given the persistent vaginal bleeding, anemia (hematocrit 14.0%, normal ≥36%), and signs of disseminated intravascular coagulopathy.

**Figure 1:**
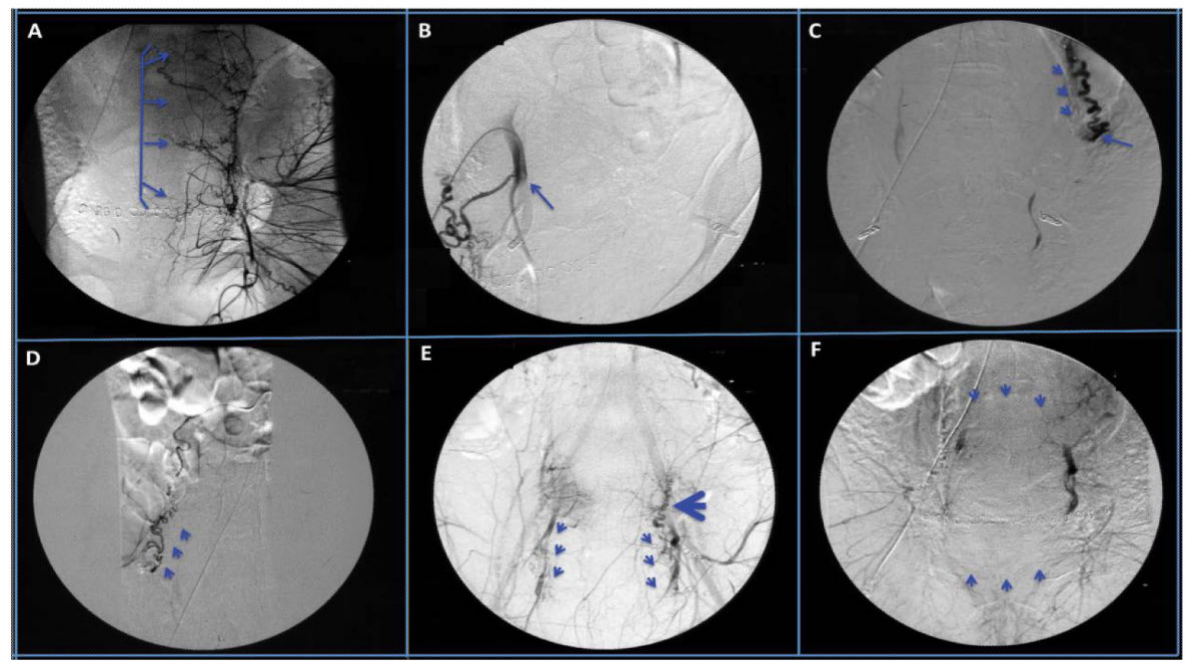
Selective angiography of the pelvic arteries demonstrating: ***A.*** Uterine branches of the left uterine artery prior to uterine artery embolization. ***B.*** Proximal origin of the right uterine artery. ***C.*** Dilated left ovarian artery seen by flushing aortogram (arrow heads), with the distal end embolized (arrow). ***D.*** Normal size right ovarian artery. ***E.*** Recanalization of the internal hypogasgric arteries (arrow heads) with and blood reflux from the uterine artery into the left ovarian artery (large arrow head). ***F.*** Post-embolization pelvic angiogram demonstrating complete cessation of blood flow to the uterus.

Gelfoam slurry and 500 to 700 micron embosphere microspheres were used throughout the procedure. Bilateral embolization of the internal hypogastric arteries (Figure 1A) was initially performed. Despite initial tamponade of the bleeding, the PPH continued. A repeat aortogram demonstrated significant persistent filling of the uterus from an aberrant right uterine artery that originated proximally from the internal hypogastric artery (Figure 1B) and a dilated left ovarian artery (Figure 1C). Due to the proximal take-off of the right uterine artery (Figure 1B), it was selectively reembolized. Both ovarian arteries (Figure 1C,1D) were also selectively catheterized and embolized to control persistent bleeding. Further pelvic angiogram demonstrated recanalization of both internal hypogastric arteries (Figure 1E), and thus both internal hypogastric arteries were re-embolized. A final aortogram demonstrated no flow from both uterine arteries, complete cessation of flow from the left ovarian artery with minimal flow from the right ovarian artery, and minimal flow from neo-vessels derived from the left external iliac artery that was non-amenable to embolization (Figure 1F). The procedure was accomplished with no complications, and the patient was discharged home on the 5^th^ postoperative day in stable condition on iron and erythropoietin therapy.

**Figure 2:**
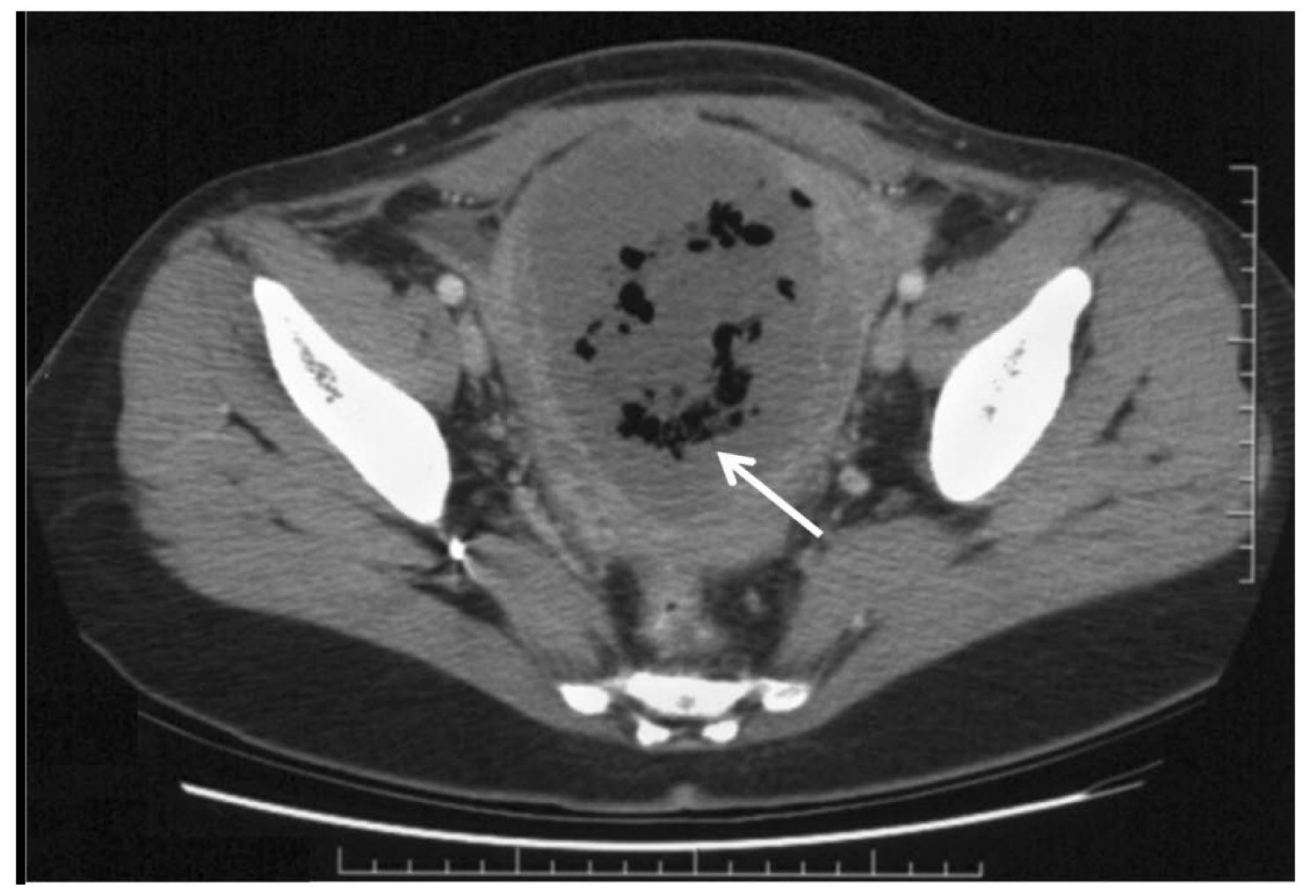
Computed tomography scan showing an enlarged uterus containing low attenuation tissue with significant amount of gas, consistent with uterine necrosis (arrow).

**Figure 3:**
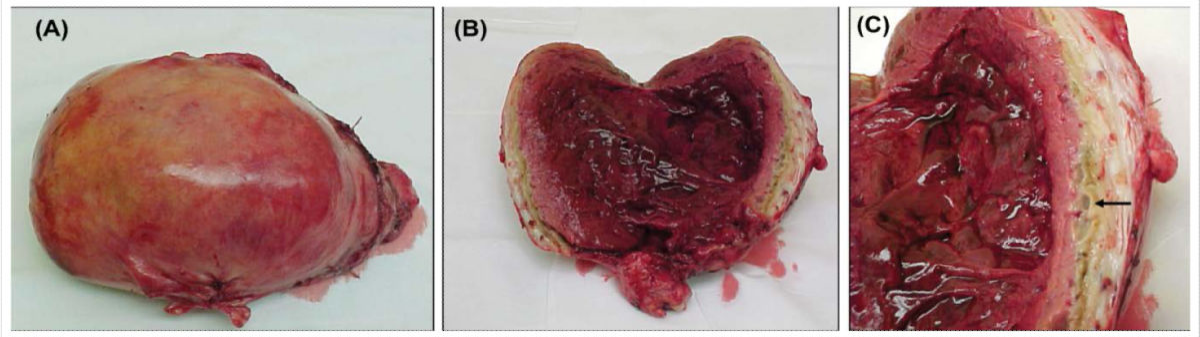
***A.*** An image of the uterus that measured 15×12×7.2cm and weighed 860grams. ***B.*** An image of the uterus, the endometrial cavity was full of blood clots. ***C.*** An image of the myometrium with necrosis and infarction of the myometrium (arrow).

**Figure 4:**
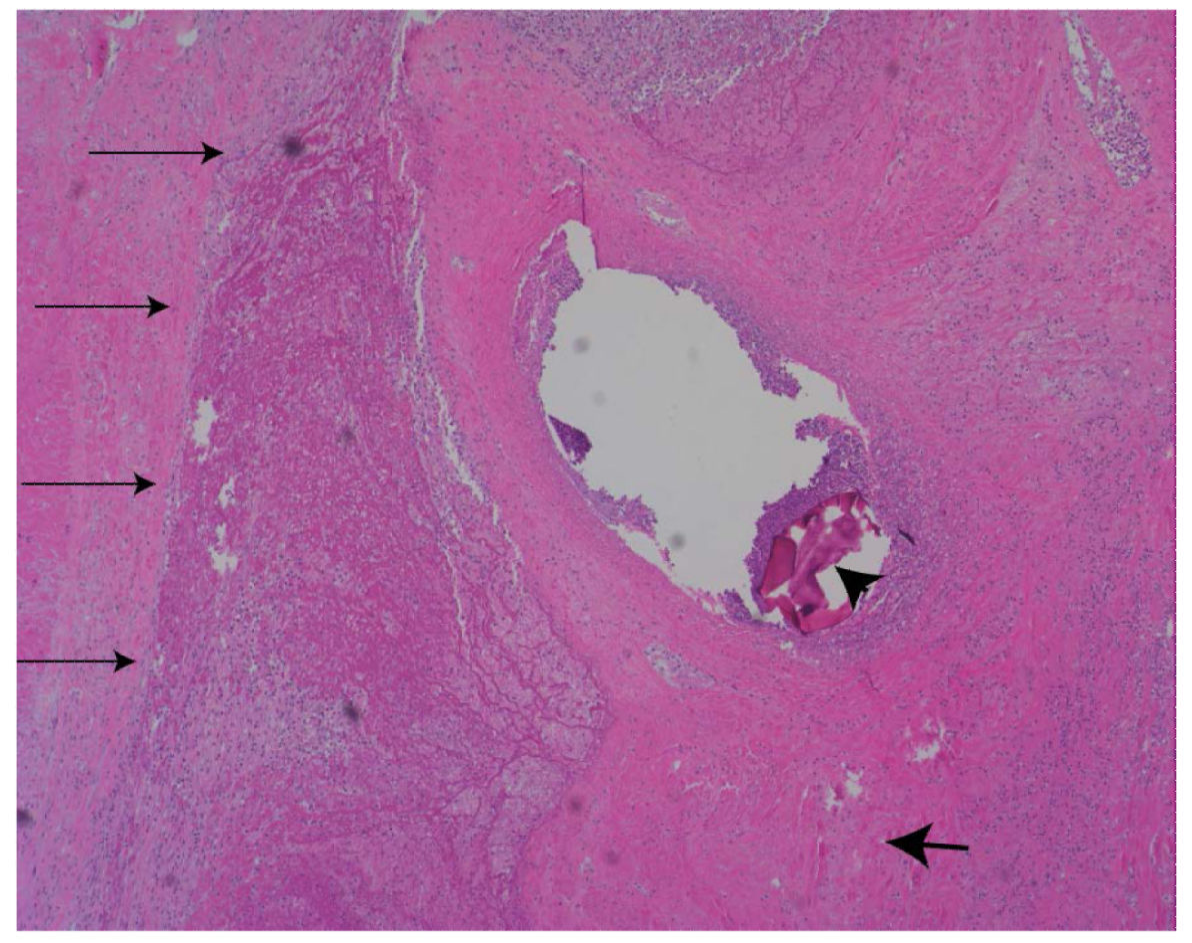
A photomicrograph of the myometrium stained with H@E demonstrating myometrial necrosis (thin arrows), embolization material (arrow head) in the blood vessels, and a multinucleated giant cell secondary to foreign body reaction (broad arrow).

Two weeks after UAE, the patient’s FSH level was elevated at 40mIU/ml, consistent with acute primary ovarian insufficiency. She was started on estrogen hormone replacement therapy. Four weeks after UAE, she presented with low-grade fevers, lower abdominal pain, and an enlarged 18-week size, tender uterus. A diagnosis of postembolization syndrome or endometritis was considered. A computed tomography scan of the abdomen revealed a large uninvoluted uterus containing low attenuation tissue with significant amount of gas raising the possibility of a necrotic uterus or an infected abscess in the uterus (Figure 2). Percutaneous aspiration of the uterine cavity collection was unsuccessful and a minimum hemorrhagic foul smelling fluid was obtained. In view of the failure of expectant management with analgesia and broad-spectrum antibiotics, an exploratory laparotomy with total abdominal hysterectomy, appendectomy, and lysis of adhesions was performed. The uterus was enlarged, soft, friable, necrotic, and foul smelling (Figure 3A-C). Both ovaries looked healthy and were conserved. Pathologic examination revealed a 17.2×12×7.2cm uterus, which weighed 860grams. The myometrium was infarcted with areas of necrosis (Figure 4). Culture of the endometrium revealed Enterococcus species. The patient had an uneventful postoperative recovery.

## Discussion

UAE is a life-saving procedure and complications are usually minimal. There is, however, a possibility of uterine infarction and subsequent ovarian insufficiency in patients with significant ovarian to uterine artery anastomoses [20]. Bleeding may continue in these patients post bilateral UAE and ovarian artery embolization may be needed. Although UAE has an advantage over surgical uterine and internal hyogastric artery ligation due to the ability to visualize, catheterize, and occlude excessive bleeding from collateral vessels, this may increase the risk of ovarian insufficiency in unique cases of aberrant pelvic vasculature when ovarian arteries are involved.

UAE may be used to address post-partum hemorrhaging with the aim of temporarily decreasing uterine blood supply while allowing time for the homeostatic system to work, without inflicting uterine and ovarian necrosis. The gravid uterus is especially vascularized and a more thorough embolization is necessary in order to achieve the same reduction in bleeding when compared to fibroid embolization. Decreasing uterine artery blood supply must be finely dialed, however, in order to prevent total ischemia and subsequent necrosis of the uterus. Normal collateral circulation from the ovarian, cervical, vaginal, vesical, and the external pudendal arteries maintain sufficient blood flow following UAE. Additional neo-vessels also often appear following embolization.

Uterine infarction has typically been reported with high injection of small-size polyvinyl alcohol particles (150-300μm), as these can migrate and block fine branches in the arterial tree, leading to ischemia [5]. Additionally, uterine infarction has been reported with ligating both the uterine and ovarian arteries during embolization [6]. In this case, embolization of uterine, ovarian, and hypogastric arteries in combination with the patient’s severe anemia and refusal of blood transfusion are suspected to have contributed to ovarian failure and the uterine necrosis. Avoiding uterine infarction may be facilitated by utilizing large size (>500μm) particles and particles with a shorter life span to allow sooner recanalization and collateral blood vessel formation. Additionally, finer micro-catheterization techniques of select collateral vessels, where the catheter tip is meticulously placed as distal as possible and reflux of embolization material is minimized, is also warranted to prevent uterine infarction [21].
